# Separating scale‐free and oscillatory components of neural activity in schizophrenia

**DOI:** 10.1002/brb3.2047

**Published:** 2021-02-03

**Authors:** Frigyes Samuel Racz, Kinga Farkas, Orestis Stylianou, Zalan Kaposzta, Akos Czoch, Peter Mukli, Gabor Csukly, Andras Eke

**Affiliations:** ^1^ Department of Physiology Semmelweis University Budapest Hungary; ^2^ Department of Psychiatry and Psychotherapy Semmelweis University Budapest Hungary

**Keywords:** electroencephalography, fractal analysis, power spectral density, scale‐free neural activity, schizophrenia

## Abstract

**Introduction:**

Alterations in narrow‐band spectral power of electroencephalography (EEG) recordings are commonly reported in patients with schizophrenia (SZ). It is well established however that electrophysiological signals comprise a broadband scale‐free (or fractal) component generated by mechanisms different from those producing oscillatory neural activity. Despite this known feature, it has not yet been investigated if spectral abnormalities found in SZ could be attributed to scale‐free or oscillatory brain function.

**Methods:**

In this study, we analyzed resting‐state EEG recordings of 14 SZ patients and 14 healthy controls. Scale‐free and oscillatory components of the power spectral density (PSD) were separated, and band‐limited power (BLP) of the original (mixed) PSD, as well as its fractal and oscillatory components, was estimated in five frequency bands. The scaling property of the fractal component was characterized by its spectral exponent in two distinct frequency ranges (1–13 and 13–30 Hz).

**Results:**

Analysis of the mixed PSD revealed a decrease of BLP in the delta band in SZ over the central regions; however, this difference could be attributed almost exclusively to a shift of power toward higher frequencies in the fractal component. Broadband neural activity expressed a true bimodal nature in all except frontal regions. Furthermore, both low‐ and high‐range spectral exponents exhibited a characteristic topology over the cortex in both groups.

**Conclusion:**

Our results imply strong functional significance of scale‐free neural activity in SZ and suggest that abnormalities in PSD may emerge from alterations of the fractal and not only the oscillatory components of neural activity.

## Graphical abstract

In this study, we separated the scale‐free (fractal) and oscillatory components of the power spectral density (PSD) of electroencephalography recordings acquired from healthy controls (HC) and patients with schizophrenia (SZ). We found increased delta band‐limited power in SZ when compared to HC in the raw PSD; however, this difference was only present in its fractal but not its oscillatory component. Our results imply strong functional significance of scale‐free neural activity in SZ and suggest that abnormalities in PSD may emerge from alterations of the fractal and not only the oscillatory components of neural activity.

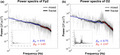

## 1 INTRODUCTION

Despite many decades of intense research, the neural origins of schizophrenia (SZ) are still mostly unknown (Uhlhaas & Singer, 2010). As a consequence, no objective biomarkers of the disease have been identified yet, which could be used for diagnosis, severity scoring or therapy and progression monitoring. One of the more potent candidates is the amplitude—or as more commonly captured, the band‐limited spectral power (BLP)—of neuronal oscillations in specific narrow‐band frequency ranges (Boutros et al., 2008). By these means, identification of abnormalities in specific frequency bands (such as delta or alpha) could imply the involvement of particular neuronal circuit architectures (Buzsaki, 2006; Javitt et al., 2020), thus providing not only markers of the disease but insights on its underlying pathomechanisms. Such approaches were able to reveal characteristic differences between patients with SZ and healthy controls (HC). Most studies report on increased amplitude of delta‐range fluctuations in SZ when compared to HC (Harris et al., 2006; Knott et al., 2001). Studies investigating normalized instead of absolute power spectra yielded similar results (Kirino, 2004; Sponheim et al., 1994), indicating that the distribution of power is shifted toward the lower frequencies in SZ. Although these findings are considered consistent by most reviews and meta‐analyses (Boutros et al., 2008; Moran & Hong, 2011; Newson & Thiagarajan, 2019), contradictory results do exist indicating that increased delta BLP is not a universal trait of SZ. Indeed, several reports (Begic et al., 2000; Harris et al., 2001; John et al., 2009) demonstrated that various disease phenotypes could be characterized with distinct EEG abnormalities in the resting state, such as decreased versus increased delta BLP in “positive” and “negative” schizophrenia, respectively. Furthermore, neuroleptic treatment (Knott et al., 2001; Matsuura et al., 1994; Tislerova et al., 2008) or disease chronicity (Harris et al., 2006; Ranlund et al., 2014) was also reported to influence electrophysiological findings in SZ, often resulting in decreased rather than increased delta activity. Finally, multiple studies identified decreased delta BLP in SZ during sleep (Keshavan et al., 1998) or associated with task performance (Bates et al., 2009; Donkers et al., 2013).

On the other hand, the limitations of treating frequency ranges independently instead of considering the power spectrum as a whole have also been stressed earlier (Moran & Hong, 2011). Specifically, it has been widely recognized that besides the narrow‐band oscillatory characteristics, neural fluctuations also express scale‐free (or *fractal*) behavior when investigated in a broadband manner (He et al., 2010). In case of scale‐free dynamics, the power is inversely proportional to the frequency in the power spectrum of the process, and the relationship is established via a power‐law function with scaling exponent *β* (Eke et al., 2002). This property is most apparent when plotting the power spectrum in double logarithmic axes, where it appears as a straight line with a slope of − *β*. In case of neurophysiological signals, oscillatory processes with characteristic frequencies (such as alpha oscillations) are found superimposed on this broadband activity; thus, an additive model considering neural activity as a composite of fractal and oscillatory components appears reasonable (He, 2014; Wen & Liu, 2016). Physiological processes other than neural activity—for example, heart rate variability (Yamamoto & Hughson, 1991)—were also shown to exhibit similar behavior. In many of these cases, the oscillatory components are in the focus of interest; however, the presence of broadband activity can distort the results of the analysis (Yamamoto & Hughson, 1991). Data processing methods such as pre‐whitening or pre‐coloring exist to deal with such issues (Bullmore et al., 2001; Mitra & Pesaran, 1999), although in general, these disregard the information encoded in the broadband component. In contrast, the physiological relevance of scale‐free brain activity has been emphasized in numerous works (e.g., He et al. (2010); Herman et al. (2011), for a review see He (2014)). Accordingly, based on the seminal works of Yamamoto and Hughson (1991, 1993), an improved analysis tool termed irregular‐resampling auto‐spectral analysis (IRASA) was developed by Wen and Liu (2016) with the explicit purpose of separating the fractal and oscillatory components in the power spectrum of neurophysiological signals. Hence, BLP of oscillatory activity can be computed without the confounding effects of broadband activity, while at the same time, the fractal signal component can be characterized by its spectral scaling exponent and/or BLP, whose estimation is not affected by the presence of oscillatory peaks.

In scale‐free processes with equal variance but different spectral slope, results similar to those found between HC and SZ individuals can be acquired. Namely, in the case of unit variance (hence unit total spectral power), a steeper spectral slope (i.e., higher scaling exponent) yields a distribution with an increased (decreased) fraction of power being associated with lower (higher) frequencies. Therefore, considering the established scale‐free nature of neural activity, it is plausible that alterations of the fractal rather than the oscillatory components could be (at least in part) accountable for increased low‐range and decreased high‐range spectral power in SZ. In this case, interpretation of such findings could be put in a different perspective, focusing also on how and why the scale‐free characteristics of neural activity are affected in SZ.

Until recently, only a limited number of studies investigated the scale‐free properties of neural activity in SZ (Nikulin et al., 2012; Sun et al., 2014). Furthermore, to the best of our knowledge, no previous study analyzed the fractal and oscillatory components of the EEG spectra separately and thus explored their contributions to BLP estimates. Therefore, the main goal of this present work was to reveal if differences in BLP found between HC and SZ individuals could be attributed to alterations of the fractal or the oscillatory components of neural activity. IRASA was utilized to separate oscillatory and fractal components of the original (mixed) power spectral density (PSD) estimates acquired from normalized EEG signals, and BLP was calculated in four frequency bands (delta, theta, alpha and beta) for all three—mixed, fractal, and oscillatory—spectra. Additionally, spectral scaling exponents of the fractal components were also estimated in order to characterize the scale‐free aspect of neural activity.

## 2 MATERIALS AND METHODS

### 2.1 Participants and data acquisition

Electroencephalography recordings of 14 SZ patients (7 females and 7 males with mean age 28.3 ± 4.1 and 27.9 ± 3.3 years, respectively) and 14 HC subjects (7 females and 7 males with mean age 28.7 ± 3.4 and 26.8 ± 2.9 years, respectively) were analyzed in this study. The datasets were acquired from an online repository made publicly available by Olejarczyk and Jernajczyk (2017a). All SZ patients met diagnostic criteria of the International Classification of Diseases ICD‐10 for paranoid schizophrenia (category F20.0) and were hospitalized at the Institute of Psychiatry and Neurology in Warsaw, Poland. Only individuals with an ICD‐10 diagnosis of category F20.0 were included in the SZ group, as well as a medication washout period of at least one week was administered for all patients prior to measurement. Exclusion criteria included age under 18 years, pregnancy, organic brain pathology, early‐stage (first onset) SZ, severe neurological diseases (e.g., epilepsy, Alzheimer's disease, Parkinson's disease) and the presence of any general medical condition. The original study was approved by the local ethics committee (Ethics Committee of the Institute of Psychiatry and Neurology in Warsaw) and all individuals provided written informed consent before participating.

EEG activity of 19 cortical regions according to the international 10–20 montage (Fp1, Fp2, F3, F4, F7, F8, Fz, C3, C4, Cz, T3, T4, T5, T6, P3, P4, Pz, O1, and O2) was recorded with a sampling rate of 250 Hz. The reference electrode was positioned at FCz. The original measurements lasted fifteen minutes and were carried out at an eyes‐closed resting‐state condition. Further details on study participants and data acquisition are found in the original article supporting the dataset (Olejarczyk & Jernajczyk, 2017b). The datasets were downloaded from the repository at http://dx.doi.org/10.18150/repod.0107441.

### 2.2 Data preprocessing

All data preprocessing steps and subsequent analyses were performed using Matlab (MathWorks, Natick, MA), while statistical analysis was done using Matlab and TIBCO Statistica 13.5 (TIBCO Software Inc., Palo Alto, CA). Data preprocessing was carried out using the EEGLAB toolbox (Delorme & Makeig, 2004) along with custom scripts. The preprocessing pipeline was designed with the intention of supporting automation at every possible step. First, all datasets were visually inspected and continuous artifact‐free segments of length at least 65 s were selected for further processing. The data segments were band‐pass filtered using a zero‐phase Butterworth filter of order 5 with lower and upper cutoff frequencies 0.5 and 45 Hz, respectively. Subsequently, artifacts of extraneural origin (i.e., eye movements, muscle contractions, and cardiac activity) were removed using the Multiple Artifact Rejection Algorithm (MARA), which is a machine learning‐based plug‐in of EEGLAB trained by professionals on thousands of EEG datasets (Winkler et al., 2011, 2014). MARA utilizes independent component analysis (ICA) to decompose EEG data into maximally linearly independent components. From these components, those that can be associated with various types of artifacts are identified based on six features capturing temporal, spatial, and spectral information (detailed in Winkler et al. (2014)) and rejected before performing reverse ICA. After artifact rejection, data were again visually inspected without knowing group labels in order to avoid selection bias, and one clean, continuous segment of length 2^14^ data points was selected from each subject for further analysis (exact positions of the final data segments used in the analysis are provided for each subject in Table S1). The data were then transformed into reference‐free Current Source Density (CSD) estimates using a spherical spline algorithm (Perrin et al., 1989). CSD transformation has the advantage over other re‐referencing schemes in providing estimates free of the actual choice of reference electrode during recording, as well as it reduces the effects of volume conduction (Nunez et al., 1997). CSD transformation was carried out in Matlab using the CSDToolbox (Kayser & Tenke, 2006a, 2006b). Finally, data from all channels were standardized in order to have zero mean and unit variance, so that their PSD estimates (see below) would yield a normalized distribution of power over frequency with the theoretical integral of the power spectrum equaling 1 (He, 2011).

### 2.3 Data analysis

#### 2.3.1 Separating scale‐free and oscillatory components in the power spectrum

**FIGURE 1:**
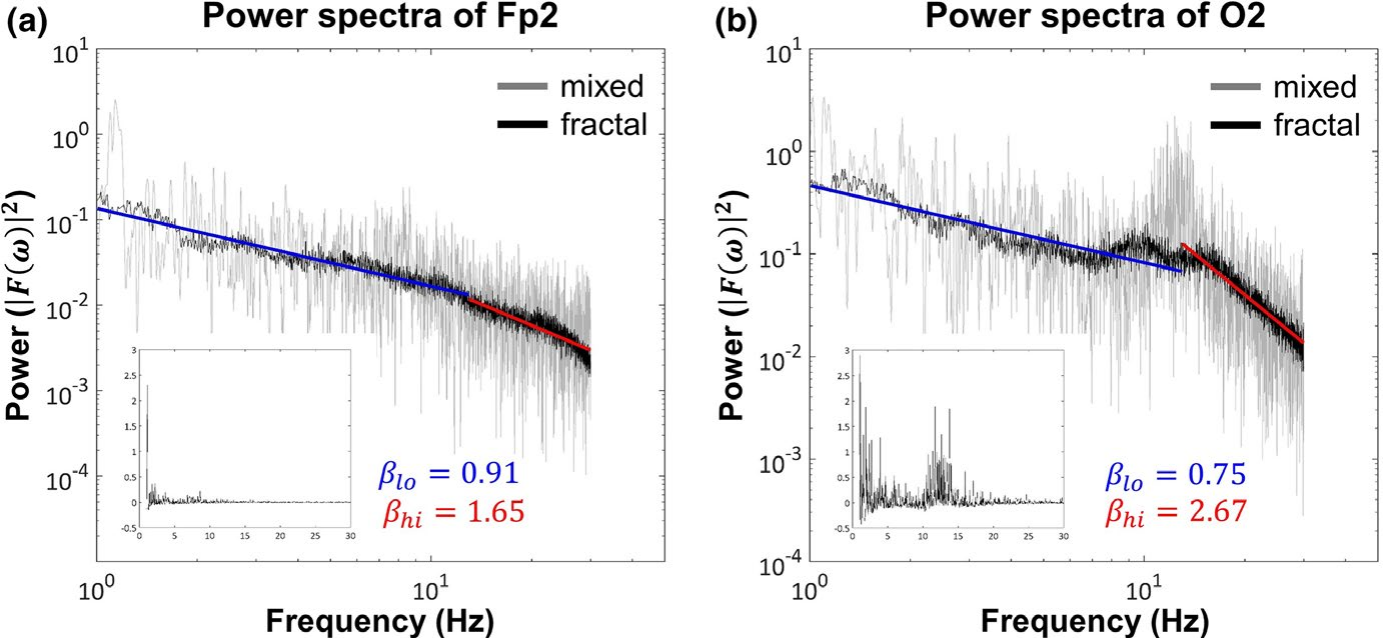
Mixed, fractal, and oscillatory spectra. Illustrative examples are shown from regions Fp2 (a) and O2 (b) with markedly different spectral characteristics. On both panels, the original/mixed PSD is marked in gray and the separated fractal component in black. The two distinct scaling ranges (low: 1–13 Hz, marked in blue; high: 13–30 Hz, marked in red) with different spectral exponents (slopes) are apparent, especially in case of O2. The inset plots show the spectra of the corresponding extracted oscillatory components, which are obtained by subtracting the fractal component from the original (mixed) spectrum. Strong alpha activity is apparent over O2 and relatively absent over F8

The Matlab implementation of IRASA as published by Wen and Liu (2016) was used to calculate the PSD estimates of the preprocessed EEG signals and to separate their scale‐free and oscillatory components (for a short summary of the theoretical basis and details of the IRASA algorithm see Appendix 1). At the utilized segment length (~65 s), it is important to consider the plausible non‐stationary nature of electrophysiological signals that might affect the IRASA analysis. Therefore, we performed Augmented Dickey–Fuller tests to check for signal non‐stationarity, which was rejected in all cases at the level α=.05. The *amri_sig_fractal* function of the IRASA toolbox was used for PSD estimation with input settings *srate* = 250, *frange*=[1, 30], *detrend* = 1, and *hset* = *linspace*(1.05, 1.5, 20). During IRASA, the PSD of the signal was estimated using fast Fourier transform with Hanning window tapering. The frequency resolution was set to be two times the smallest power of 2 that was greater than the number in the resampled data segments. The resampling scheme was applied using resampling factor pairs *h* and 1/*h* with 20 values of *h* evenly distributed between 1.05 and 1.5. The maximum value of the resampling factor *h* was set to 1.5 so that resampling would not introduce a filtering effect in the range 1–30 Hz (for more details, see Supplementary Material). Moreover, in order to provide more reliable estimates, IRASA returns the mean PSD obtained from 15 overlapping segments of the original data, each with size 90% of that of the total signal length. The outputs of IRASA, namely the mixed PSD, and the fractal and oscillatory components are illustrated in Figure 1. Note that IRASA estimates the power spectral density that is not strictly equivalent to the power spectrum (Miller & Childers, 2012); however, for the sake of simplicity, in the following we will refer to the PSD estimates and their fractal and oscillatory components as mixed, fractal, and oscillatory spectra.

#### 2.3.2 Band‐limited power and spectral slope calculation

We investigated band‐limited power (BLP) of the mixed, fractal, and oscillatory components in four frequency bands traditionally used in EEG analysis: delta (1–4 Hz), theta (4–8 Hz), alpha (8–13 Hz), and beta (13–30 Hz). BLP was acquired as the sum of power (squared absolute amplitude) within the given frequency range.

Spectral exponent (*β*) estimation of the fractal component for each channel was carried out using the *amri_sig_plawfit* function of the IRASA toolbox. In that, the spectral slope is acquired by fitting a power‐law function on the fractal power spectrum. This is achieved by first log‐log transforming frequencies and their corresponding powers. However, this procedure results in an overrepresentation of higher frequencies; therefore, frequencies are resampled to yield an even representation on the logarithmic scale. Then, least‐squares regression is used to obtain the best fitting linear function, whose slope gives the spectral exponent *β* of the power spectrum.

It has been shown previously that neurophysiological signals can express a multimodal nature that is, they have multiple distinct scaling ranges with different spectral slopes in their power spectra (He et al., 2010; Nagy et al., 2017; Wen & Liu, 2016). In that, the power spectrum can be divided into a slow component ranging approximately from 1 to 10 Hz with a smaller, and a high‐frequency component with a steeper spectral slope. With sufficient temporal resolution and measurement length, further ultraslow (below 0.5 Hz) and ultrahigh (approximately over 50 Hz) regimes can be separated (for details see He et al. (2010) and Wen and Liu (2016), respectively). The data analyzed in this study allowed for reliable spectral estimates in the 0.5–30 range, thus we treated neural signals as bimodal, and defined the slow component as ranging between 1 and 13 Hz and the fast component as ranging from 13 to 30 Hz. Consequently, the spectral slope was calculated in these two frequency ranges separately, yielding estimates of *β*_lo_ and *β*_hi_ characterizing the slope of the fractal power spectrum in the 1–13 and 13–30 Hz regimes, respectively (see Figure 1). The boundary frequency was defined as 13 Hz in order to provide consistency among BLP and spectral exponent analyses.

It is important to emphasize that we worked with standardized time series in this study. Since the total integrated power of the power spectrum yields the variance of the signal (which in the standardized case is equal to 1), this means that BLP estimates in this study reflect on the relative distribution of power among frequencies instead of absolute power. On the other hand, standardization has no effect on the spectral slope itself. Furthermore, standardization also yielded normally distributed BLP estimates in most cases. In many studies, normality of the data is ensured by log‐transforming the absolute (i.e., non‐normalized) BLP estimates (see e.g., Kam et al., 2013). However, during IRASA, estimates of the oscillatory spectrum are acquired by subtracting the fractal spectrum from the mixed spectrum and thus this procedure can yield negative values preventing log‐transformation.

### 2.4 Statistical analysis

#### 2.4.1 Channel‐wise analysis

Band‐limited power estimates in all four frequency ranges as well as *β*_lo_ and *β*_hi_ values were compared between HC and SZ subjects in a channel‐wise manner. In that, Lilliefors tests were applied first to verify normality of the data. If either group failed at this step, a Mann–Whitney U test was used for group comparison. Otherwise, *F* test was used to confirm equality of variances in the two groups, and Welch‐corrected *t* test was applied in case of unequal variances while a two‐sample *t* test otherwise. Finally, the false discovery rate (FDR) method of Benjamini and Hochberg (1995) was applied to control for multiple comparisons with level α=.05. For all significant differences, we also computed the adjusted power (AP) and the effect size (ES) in TIBCO Statistica. Also, in order to verify that spectral exponents of low‐ and high‐range neural activity are indeed different (i.e., the EEG data have a bimodal PSD), we tested if the differences between *β*_lo_ and *β*_hi_ acquired as $\Delta \beta =\beta_{\text{hi}}‐\beta_{\text{lo}}$ are significantly different from zero for every channel. In that, we used one‐sample *t* tests or one‐sample Wilcoxon signed rank tests (in case of non‐normal distribution of Δβ as confirmed by Lilliefors test) separately for HC and SZ groups and applied FDR correction with level α=.05 to control for multiple comparisons. Furthermore, in order to confirm that a bimodal model provided a better fit for the power spectra than a unimodal model (estimating a single *β* utilizing the entire 1–30 Hz range), Goodness‐of Fit (GoF) statistics obtained with the two approaches were compared using *F* tests (for details, see Supplementary Material).

#### 2.4.2 Resting‐state network analysis

**FIGURE 2:**
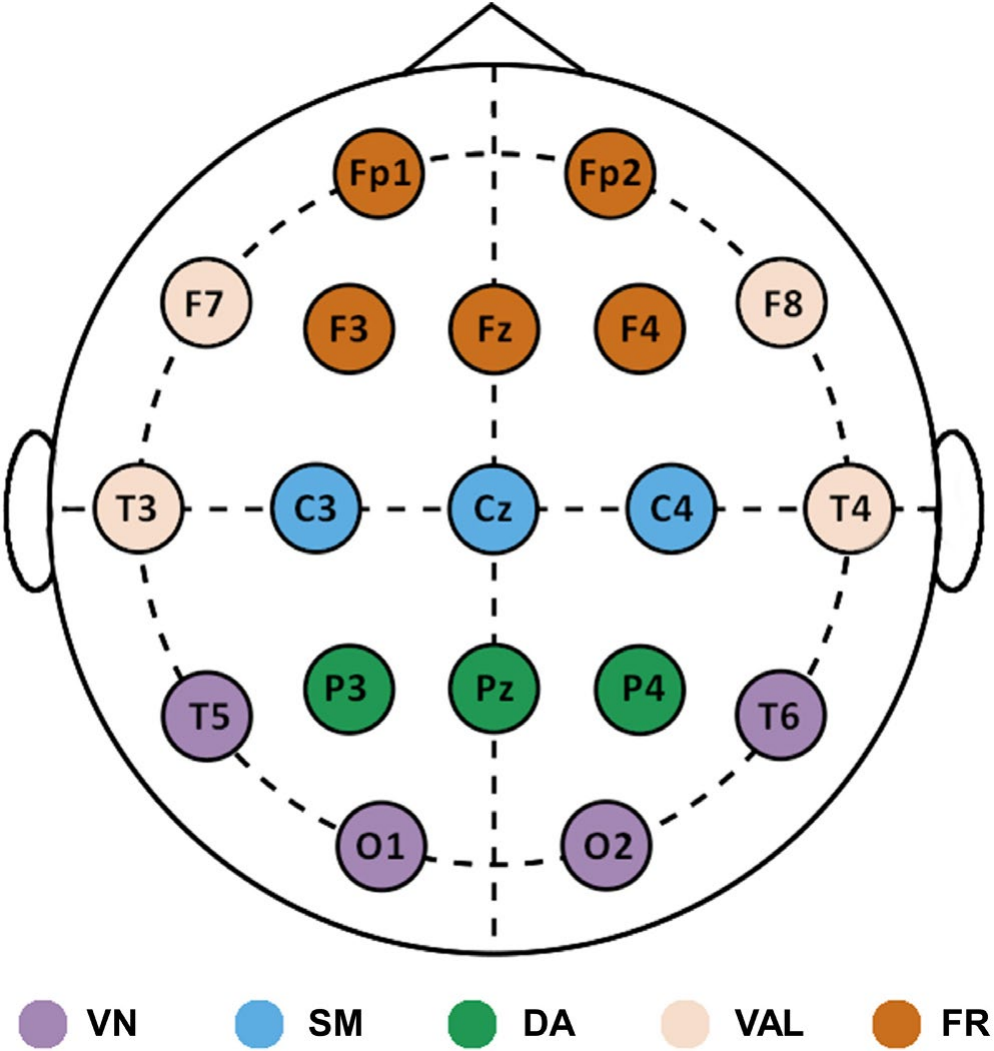
Electrode layout and resting‐state networks. The parcellation reflects the functional organization of the brain. The five RSNs are marked in different colors. RSN = resting‐state network; VN = visual network; SM = somatomotor; DA = dorsal attention; VAL = ventral attention‐ and limbic; FR = frontal

In order to reduce dimensionality of the results, we grouped the channels according to which intrinsic functional network of the brain they most likely represent. This procedure was carried out following the probability maps provided in Giacometti et al. (2014), similarly as in a previous study (Racz et al., 2019). Brain parcellation was performed so that channel groups represented seven intrinsic resting‐state networks (RSN) of the brain, as identified by Yeo et al. (2011). Note that here under the term “resting‐state network,” we refer to a collection of brain regions that were identified as functionally coupled based on functional magnetic resonance imaging studies. Therefore, grouping of the channels was carried out so that groups reflect the functional organization of the brain. With a limited spatial resolution of 19 channels, some regions could not be unequivocally assigned to one RSN. Thus, in two cases we grouped channels to represent the joint activity of two RSNs, resulting in a final number of five groups. These included the visual network (VN, channels O1, O2, T5, and T6), the somatomotor network (SM, channels C3, C4, and Cz), the dorsal attention network (DA, channels P3, P4, and Pz), the combined ventral attention and limbic networks (VAL, channels F7, F8, T3, and T4), and a joint frontal network (FR) comprising regions of the frontoparietal (channels F3 and F4) and the default mode networks (channels Fp1, Fp2, and Fz). The channel groups representing the five RSNs are shown in Figure 2. Similarly to channel‐wise analysis, BLP estimates of the mixed, fractal, and oscillatory spectra in all five frequency bands along with low‐ and high‐range spectral exponents were investigated. For each case, the given index for a particular RSN was acquired by averaging the values over the channels belonging to that RSN. During the RSN‐level analysis, between‐group differences of corresponding networks were investigated according to the same statistical principles as in channel‐wise analysis.

## 3 RESULTS

### 3.1 Low‐ and high‐range spectral exponents

**FIGURE 3:**
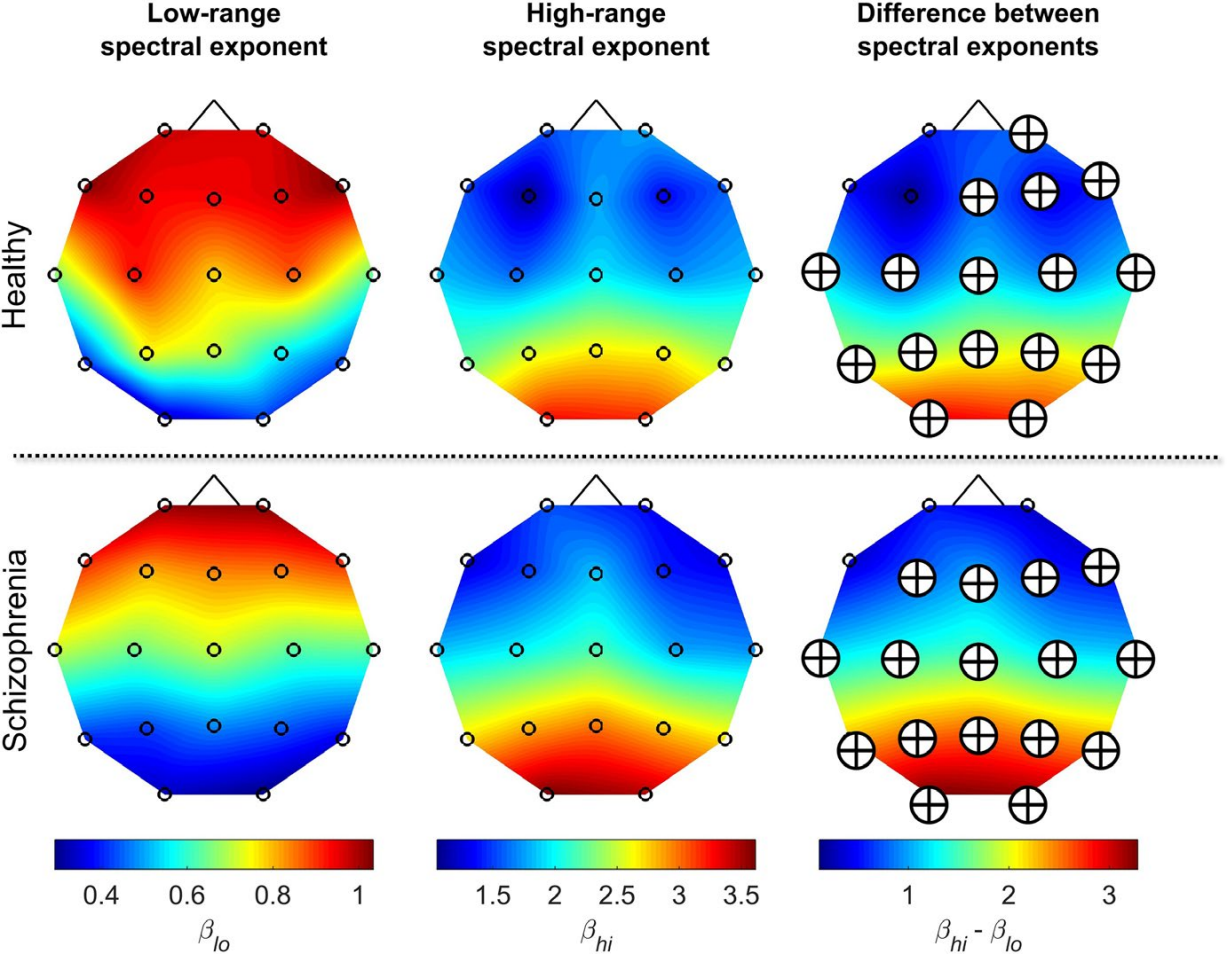
Topology of spectral slopes. Group‐averaged spatial maps of *β*_lo_ (left) and *β*_hi_ (middle) reveal characteristic topologies in both groups. Regions where the difference between high‐ and low‐range spectral slopes (right) was found significantly different from 0 following FDR adjustment with level α=.05 are marked with crossed circles

A characteristic spatial distribution of *β*_lo_ and *β*_hi_ was observable over the cortex in both groups (Figure 3). In that, *β*_lo_ was higher over the frontal and central regions, while the opposite topology was revealed in *β*_hi_ with the highest values observed over the occipital cortex. Although a tendency of lower *β*_lo_ over the central regions could be observed in SZ subjects (see left panels of Figure 3), no significant difference was found between HC and SZ groups following FDR adjustment Δβ was found significantly different from zero (*p* < .05 in all cases, corrected) over 16 out of the 19 investigated cortical regions in both HC and SZ groups (Figure 3, right). Notably, Δβ was found smaller over the frontal when compared to occipital regions in both groups, as well as fractal spectra were found unimodal over the Fp1, F3, and F7 regions in the HC and over Fp1, Fp2, and F7 regions in the SZ group. Furthermore, comparing GoF statistics of uni‐ and bimodal fits also indicated that the latter provided a better characterization of the power spectra in the vast majority of cases (see Table S3), while regions where the power spectrum was found rather unimodal corresponded well with those where no difference was found between *β*_lo_ and *β*_hi_. Nevertheless, these results indicated a truly bimodal nature of scale‐free neural activity.

It is important to note, that in our analysis, we utilized segments of length ~ 65 s, which is considerably longer than the window sizes (3–10 s) used in previous IRASA‐based studies (Kolvoort et al., 2020; Muthukumaraswamy & Liley, 2018; Wen & Liu, 2016). Therefore, we re‐analyzed our datasets using three additional (2.5, 5, and 10 s) window sizes. In this analysis pipeline, for each window size we obtained spectral slopes from 100 consecutive, overlapping data segments with a displacement of 0.5 s, and statistically evaluated the likelihood that the spectral slopes acquired when using the entire signal came from the same distribution as those obtained with smaller sliding windows (for results, see Supplementary Material). Results obtained from this analysis showed that for all window sizes, the original spectral slopes were representative of the populations obtained with smaller time windows in almost all cases (Table S2), indicating that window size did not have a substantial effect on the results.

### 3.2 Channel‐wise results of mixed, fractal, and oscillatory BLP

**FIGURE 4:**
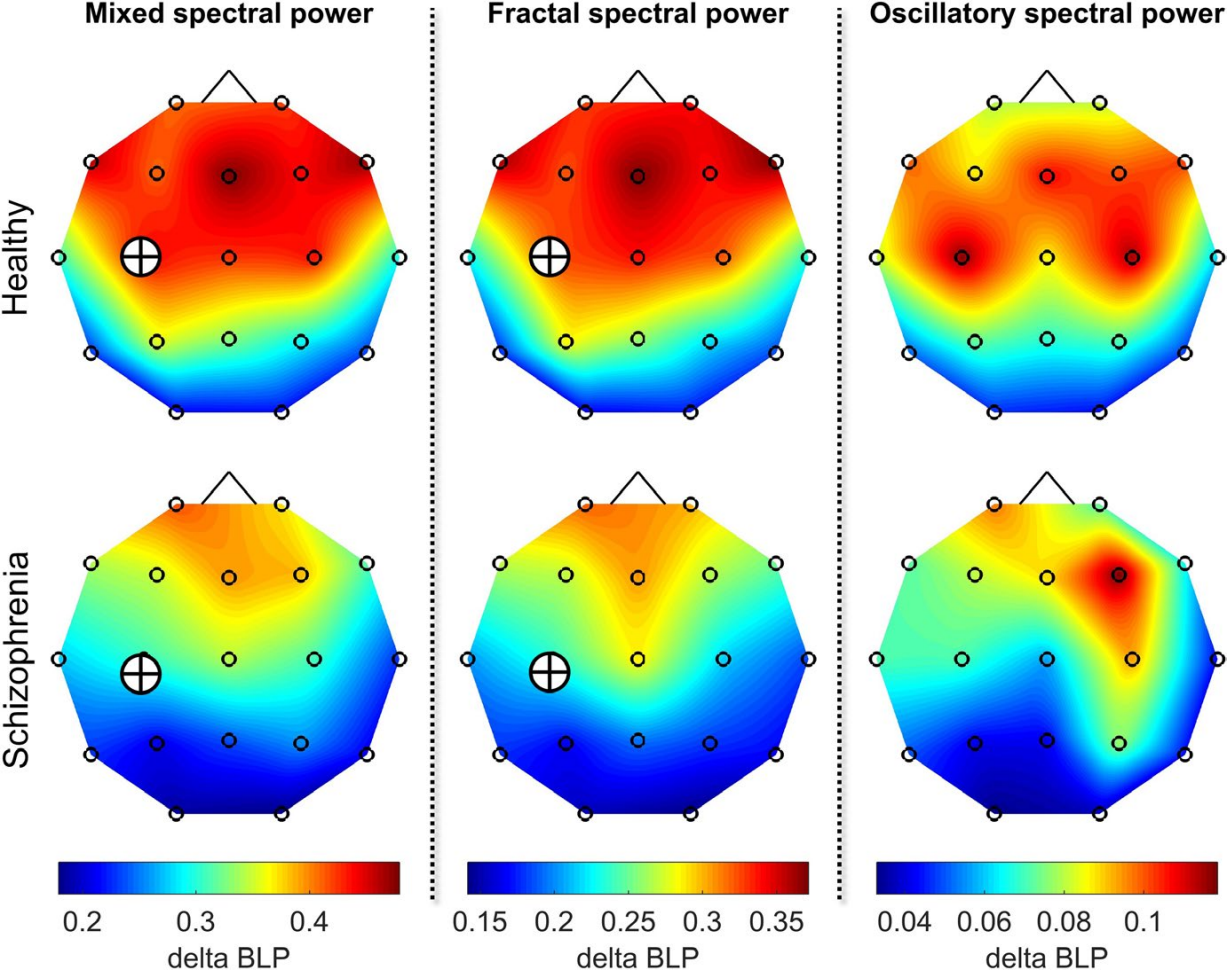
Topology of delta‐band BLP. Group‐averaged delta‐band BLP maps of the mixed (left), fractal (middle), and oscillatory (right) spectra of HC and SZ groups reveal stronger relative delta power over the frontal and central regions. The corresponding group‐average spatial maps are on the same scale for better comparison demonstrating the higher values in HC, especially in case of mixed and fractal spectra. Crossed circles mark between‐group differences that were found significant following FDR adjustment with level α=.05. HC = healthy control; SZ = schizophrenia; BLP = band‐limited power; FDR = false discovery rate

Significant between‐group differences were found only in the delta band (Figure 4). In that, HC subjects expressed significantly higher delta BLP in the mixed spectrum over the C3 (*p* = .0371, corrected, AP = 0.3620, ES = 1.0994). The same difference was found when investigating the fractal component of the power spectrum (*p* = .0433, corrected, AP = 0.3415, ES = 1.0764). On the other hand, no significant between‐group difference was found in oscillatory delta BLP following FDR adjustment. Furthermore, in order to verify that the difference observed in mixed BLP could at least in part attributed to differences in fractal BLP, we performed analysis of covariance (ANCOVA) in which the effect of group (HC vs. SZ) was investigated on mixed BLP with fractal BLP included as a covariate. The inclusion of fractal BLP in the model rendered the main effect of group in mixed BLP non‐significant (*p* = .3354), confirming that the significantly lower delta BLP over C3 in HC was at least in part a consequence of altered fractal BLP.

### 3.3 RSN‐level results of mixed, fractal, and oscillatory BLP

**FIGURE 5:**
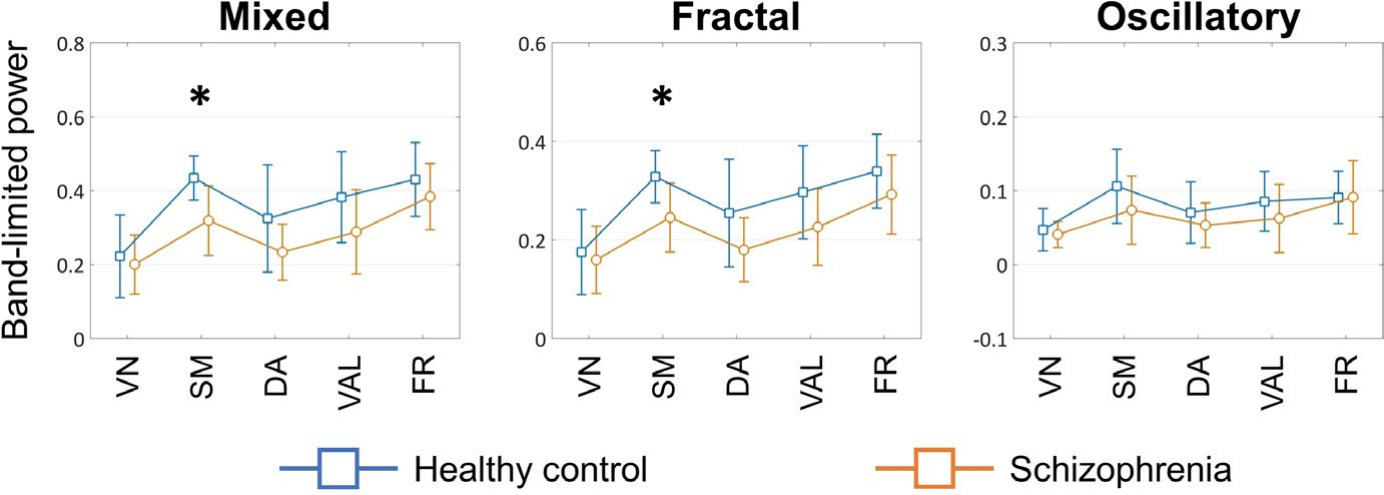
Between‐group differences in corresponding RSNs in delta‐band BLP. Asterisk symbols mark differences that were found significant following FDR correction with level α=.05. RSN = resting‐state network; BLP = band‐limited power; FDR = false discovery rate; VN = visual network; SM = somatomotor; DA = dorsal attention; VAL = ventral attention‐ and limbic; FR = frontal

The characteristic differences could be captured more robustly when channels were collapsed onto RSNs to better represent the functional organization of the brain (Figure 5). Accordingly, mixed and fractal delta‐band BLP were found significantly higher in HC subjects over the SM network (*p* = .0035, AP = 0.6384, ES = 1.1832 and *p* = .0079, AP = 0.5761, ES = 1.1174 for mixed and fractal BLP, respectively, corrected), while no differences were found in oscillatory BLP between the two groups. ANCOVA analysis showed that including fractal BLP as a covariate renders the observed difference in mixed BLP non‐significant (*p* = .1761), indicating that the lower delta BLP over the SM network in SZ was at least in part due to lower fractal BLP. Similarly to channel‐wise results, no differences were found in the theta, alpha, or beta bands.

### 3.4 Validation of the results

Due to the frequency range (0.5–45 Hz) of the preprocessed signals, we were restrained to utilize a smaller set of resampling factors extending from 1.05 to 1.5. Although these settings allowed for a broader effective frequency range in estimating the fractal component of the spectrum and a well‐defined breakpoint between the low‐ and high‐range regimes, they came at the expense of occasionally imperfect elimination of large oscillatory components such as a broad alpha peak (Wen & Liu, 2016). Therefore, it was crucial to verify the observed differences using a broader set of resampling factors, where spectral slope and fractal/oscillatory BLP estimation are less likely to be biased. For this purpose, we re‐analyzed all datasets with *h* ranging from 1.05 to 2.0 (25 evenly distributed values). As *h* = 2 limits the effective frequency range to 1–22.5 Hz, in this analysis we only considered *β*_lo_ and BLP values from the delta, theta, and alpha bands. Results obtained from this analysis pipeline were found well in line with those obtained with *h* ranging from 1.05 to 1.5, with the exception that the difference in fractal BLP between HC and SZ over C3 was found only marginally significant (*p* = .0663, corrected, AP = 0.3158, ES = 1.0469). Further details of this approach and the acquired results are provided in the Supplementary Material.

## 4 DISCUSSION

In this study, we applied a novel tool for the analysis of resting‐state EEG acquired from schizophrenic patients and healthy controls, namely separating the scale‐free and oscillatory components of their neurophysiological recordings using IRASA (Wen & Liu, 2016). Our analysis revealed decreased delta BLP in patients with SZ; however, the differences found in the original (mixed) spectra could be attributed to alterations in the fractal rather than the oscillatory component. Electrophysiological activity in both groups was confirmed to have a bimodal PSD over most cortical regions in accordance with previous studies (He et al., 2010; Nagy et al., 2017). Additionally, we found marked spatial variability of scaling exponents in both groups, further highlighting the importance of the proposed approach.

Surprisingly, our results indicated a shift toward higher frequencies in the distribution of spectral power in SZ patients, leading to a decrease of delta BLP over the central regions. This is in contrast with consistent findings of increased delta activity frequently reported in schizophrenic patients (for a recent review, see Newson and Thiagarajan (2019)). There are numerous factors that could lead to these seemingly contradictory results. Probably, the most general cause is the fundamentally heterogeneous nature of schizophrenia in terms of widely varying symptomatology, affected psychocognitive functions and disease severity (Moran & Hong, 2011; Seaton et al., 2001). Accordingly, several studies specifically attempted to resolve the inconsistencies regarding quantitative EEG analysis in SZ. Begic et al. (2000) investigated the effects of disease phenotype (i.e., positive or negative), diagnostic criteria and medication on EEG findings in SZ. They found a sharp contrast between negative and positive phenotypes, with the former characterized by an increase in delta, theta, and beta, and a decrease in alpha activity, while the latter with both decrease and increase in delta activity. Their results are in accordance with those of Saletu et al. (1990), who also reported increased and decreased delta activity in SZ patients with mainly negative and positive symptoms, respectively. Furthermore, the shift toward higher frequencies, as captured in increased beta activity, was more pronounced in the positive than in the negative SZ group (Saletu et al., 1990). John et al. (2009) reported higher alpha BLP in SZ patients with positive symptoms, while also suggested that an increase in delta activity is linked to negative symptomatology spanning from hypometabolism of the frontal cortical regions. Harris et al. (2001) sorted SZ patients into three groups based on their psychopathological symptoms and reported that while the “disorganization syndrome” and “psychomotor poverty syndrome” subtypes could be characterized with higher delta, theta and lower alpha activity, the “reality distortion” group was characterized with increased alpha activity. On a different note, it is well established that the acute psychotic phase of SZ is predominantly characterized by positive symptoms (i.e., attention deficit, reality distortion, agitation, anxiety) and hyperdopaminergia; while in chronic, medicated SZ negative symptoms (cognitive deficit, decreased motivation, blunted affect, social withdrawal) are more common (Laruelle et al., 1999; Sponheim et al., 2010; Wang et al., 2013). Accordingly, electrophysiological differences between the various phases of SZ might be expected. Indeed, several studies have found that augmented delta and theta activity could only be observed in chronic but not first‐episode or early‐stage SZ (Harris et al., 2006; Ranlund et al., 2014). These results, however, are also challenged by studies reporting no difference between first‐episode and chronic SZ (Sponheim et al., 1994) or finding elevated delta and theta activity in first‐episode patients (Clementz et al., 1994; John et al., 2009). Pharmaceutical treatment is also frequently reported to introduce alterations in the EEG spectra, usually resulting in a slowing of cortical rhythms (Harris et al., 2006; Itoh et al., 2011; Knott et al., 2001; Tislerova et al., 2008). Nevertheless, medication effects are unlikely to influence the results presented here, as subjects went through a medication washout period prior to measurement. Finally, another reason behind the controversies could be that some studies worked with non‐normalized, while others with normalized power spectra (Newson & Thiagarajan, 2019), although this seems unlikely as generally similar results can be acquired when applying both methods (John et al., 1994). Without clinical data regarding symptomatology, medication history and disease duration of SZ subjects on hand, the findings of decreased delta BLP reported in our study cannot be fully explained or linked to symptoms of schizophrenia and require further research. With the above considerations in mind, the most plausible explanation for our results is that the patient cohort consisted of young subjects characterized with positive symptomatology and free of drug‐related effects due to the medication washout period prior to measurement, although in absence of medical data, this explanation remains speculative. Nevertheless, our data analysis pipeline was designed to be maximally data driven and thus readily reproducible with the exact same settings on different datasets with the necessary clinical information supplied, thus hopefully facilitating further research aiming at resolving this issue.

Many previous studies reporting on EEG abnormalities implicitly considered narrow‐band neural activity emerging from neuronal circuit mechanisms characteristic of various cortical areas (Buzsaki & Draguhn, 2004; Javitt et al., 2020). Consequently, findings were mostly implemented as reflecting the involvement of specific brain regions responsible for generating such rhythmic activity. In that, elevated delta activity was often seen as resulting from the aberrant function of thalamocortical projections (Hunt et al., 2017; Llinas et al., 1999). Aberrations in alpha BLP are also frequently associated with the dysfunction of the thalamus and its role in cortical synchronization (Goldstein et al., 2015; Kirino, 2004). In addition, both delta and alpha activity have been associated with a generalized decline in the function and metabolism of the frontal cortex, that is, hypofrontality (Gattaz et al., 1992; Knott et al., 2001; Knyazeva et al., 2008). Many of these conclusions are well in line with results acquired by utilizing source reconstruction approaches allowing for identification of affected brain regions (Kim et al., 2015; Mientus et al., 2002; Pascual‐Marqui et al., 1999). Furthermore, they are also supported by evidence from studies using different imaging techniques with exact spatial localization, such as positron emission tomography or functional magnetic resonance imaging (Andreasen et al., 1997; Damaraju et al., 2014; Wolkin et al., 1992). On the other hand, the findings reported here indicate that EEG differences between HC and SZ subjects could not be attributed solely to alterations of the rhythmic (oscillatory), but necessarily to the arrhythmic (broadband) component of neural activity, too. This hypothesis is supported by the fact that when we separated the oscillatory and fractal components of neural activity, BLP differences found in the mixed spectra were present only in the fractal but not in the oscillatory components. Furthermore, when we included fractal BLP as a covariate into the analysis of mixed BLP, it rendered the previously observed differences non‐significant, further indicating that reduction of mixed BLP in SZ can be attributed (at least in part) to a reduction in fractal BLP. In addition, both fractal BLP and spectral slopes revealed significant spatial variability over the cortex, indicating that scale‐free brain activity indeed has functional significance (as discussed below) instead of merely being noise (He et al., 2010). These findings raise the possibility that involvement of different functions and mechanisms, namely those generating the scale‐free component of neural activity, may also play an important role in the neural basis and pathomechanism of SZ.

There has been a considerable debate on the role and functional significance of scale‐free brain activity. In fact, since scale‐free dynamics are ubiquitously present in a plethora of natural processes (Per Bak, 1996; Brown et al., 2002; Gisiger, 2001; Mandelbrot, 1983), in many cases, the fractal component of neural activity is discarded from analysis and referred to as “1/*f* noise” (Mitra & Pesaran, 1999; Zarahn et al., 1997). On the other hand, there has been growing evidence lately pointing to the direction that scale‐free brain activity carries substantial functional significance and contains fine temporal structuring that differentiates it from other natural phenomena expressing fractal dynamics (He et al., 2010). It has been shown that the scaling exponent of global neuronal synchronization in alpha and beta activity decreases when transitioning from eyes‐open to eyes‐closed states (Racz et al., 2018; Stam & de Bruin, 2004). The spectral slope was also reported to reduce during increased cognitive performance (Ciuciu et al., 2012; He, 2011; He et al., 2010; Zilber et al., 2012). As a higher (lower) spectral slope indicates stronger (weaker) autocorrelation, this change may reflect a required switch of the brain to more efficient online information processing during task solving (He, 2011). This is in line with reports of lower spectral slope in adults with trait anxiety (Tolkunov et al., 2010) indicating a constantly active state. As anxiety is often a core feature of schizophrenia (Muller et al., 2004), a lower spectral exponent of brain activity could be expected in patients. Indeed, lower spectral slope (Radulescu et al., 2012) and reduced fractal dimension and autocorrelation (Bullmore et al., 1994) were observed in SZ subjects, in accordance with our results indicating a tendency of lower *β* in SZ. It has to be noted however that the data analyzed in this study were obtained in a resting state; therefore, further research is required in order to draw conclusions on the interrelatedness of scale‐free brain activity, cognitive performance, and schizophrenia. Since power‐law scaling is a characteristic feature of critical systems operating near a phase transition (Stanley, 1971), scale‐free neural—even in the resting state—activity is also often considered as an indicator of an underlying self‐organized critical state (Bak et al., 1987) of brain function (Bullmore et al., 2009; Chialvo, 2004; Linkenkaer‐Hansen et al., 2001; Racz et al., 2018). According to this theory, criticality would provide an optimal state for the brain to quickly perform large‐scale reorganizations in response to stimuli and thus efficiently adapt to changes in the external and/or internal environment (Bullmore et al., 2009; Kitzbichler et al., 2009). In this framework, alterations of scale‐free neural activity may reflect inadequate processing of incoming sensory stimuli, a hypothesis in line with those suggesting dysfunctional information processing in SZ (Barrett et al., 1986; Callaway & Naghdi, 1982; Carr & Wale, 1986). Scale‐free properties of brain activity and neuronal synchronization were also reported to vary significantly over different cortical regions (He, 2011; He et al., 2010; Racz et al., 2019; Wink et al., 2008). Concordantly, we found relatively lower *β*_lo_ and higher *β*_hi_ over the visual and dorsal attention networks when compared to other RSNs in both groups. The spectral exponent of neural activity was also found associated with self‐consciousness (Huang et al., 2016; Kolvoort et al., 2020) and contextual prediction (Dave et al., 2018), two higher order brain functions related to top‐down cognitive processing and often affected in SZ. Spectral slope was also found reduced in elderly when compared to young subjects (Mukli et al., 2018; Voytek et al., 2015). A hypothesis that could partially explain these results suggests that broadband scale‐free neural activity emerges regionally from the spatial integration of asynchronous spiking of neuronal populations (Miller, 2010; Miller et al., 2014) and thus a reduction in *β* reflects further functional decoupling (He et al., 2010). This correspondence of neuronal synchrony and scale‐free neurodynamics also extends to macroanatomical brain networks, as the regional variability of scale‐free neural dynamics was shown to positively correlate with the large‐scale functional connectivity of brain regions (Anderson et al., 2014; Baria et al., 2013; Ciuciu et al., 2014; Radulescu & Mujica‐Parodi, 2014). Furthermore, simulations with self‐organized critical systems indicate that the fractal scaling property might also be related to the size of coupled neuronal assemblies producing scale‐free dynamics, that is, the scaling exponent of local neuronal fluctuations may reflect incoming signaling (local connectivity) to the investigated brain region (Mukli et al., 2018). Since alterations of functional connectivity are evident in schizophrenia (van den Heuvel & Fornito, 2014), a better understanding of the scale‐free component of neural activity may also provide further insights on how and why brain networks are affected in SZ. With these considerations in mind, although our findings obtained here are in contrast with those most commonly reported in the literature, we tentatively propose that alterations of a different nature (i.e., enhanced delta activity) could also be partially explained by dysfunction in scale‐free brain activity and its corresponding cognitive functions as discussed above. It has to be stressed once again, however, that unfolding the plausible relationship between scale‐free neural activity and cognitive functions/information processing in SZ requires more elaborate research paradigms. Therefore, the approach introduced here might provide a useful tool to further the understanding and implementation of EEG spectral findings in SZ.

Finally, we have to address the limitations of this study alongside its future perspectives. Foremost, we could not explore the plausible correlations between our findings and clinical features of SZ due to the lack of supporting clinical data. Thus, some of the conclusions drawn in this study remain elusive until further validation on a patient cohort with available clinical details regarding symptom scores, disease duration and medication history. This is also required for exploring the potentials in fractal measures of brain electrical activity as future biomarkers of schizophrenia. Note however that our main goal here was to explore if the scale‐free component of neural activity carries functional significance in SZ, which could be achieved despite this limitation. The small sample size also poses a drawback by limiting the statistical power of the results; therefore, a re‐evaluation of this pipeline operating on a larger group of subjects is desirable. This latter statement is indeed relevant considering that multiple between‐group differences (such as lower *β*_lo_ or higher fractal theta) were found initially significant but were then rendered non‐significant by FDR adjustment. The samples analyzed in this study were recorded in a resting‐state, eyes‐closed condition. Although this experimental setup has several advantages such as measurements are less corrupted by artifacts originating from blinking, eye or muscle movement, and that the protocol requires minimal cooperation from the subject, it also has some drawbacks in that mental processes and self‐referential activities are unconstrained in resting‐state, which can introduce a substantial bias to the results (Miall & Robertson, 2006; Weinberger & Berman, 1996). This can be of particular importance in the case of schizophrenia, where not only these processes are generally distorted, but also show a great variability between disease phenotypes (Sass & Parnas, 2003). On the other hand, scale‐free brain activity was known to be modulated by cognitive task performance (Ciuciu et al., 2012; He, 2011; He et al., 2010; Zilber et al., 2012); therefore, an experimental design including a cognitive stimulation paradigm that would allow for investigating if this modulation is affected in SZ seems promising. In this study, we analyzed continuous EEG recordings of length ~65 s. This epoch length is considerably longer than what is used in most studies, usually ranging between 2 and 30 s (Boutros et al., 2008). Moreover, only one segment per subject was analyzed; however, it is recommended to derive estimates based on an ensemble of epochs (Boutros et al., 2008). This latter issue was partially resolved, as IRASA per se calculates the PSD estimates from 15 overlapping data segments to provide robust statistics (Wen & Liu, 2016). We also chose to work with longer segments in order to have sufficient representation of low‐frequency components. It is also known that even in the resting state, fractal properties (such as *β*) of neural activity may change over time (Wen & Liu, 2016). In other words, the scaling property itself becomes a *local* instead of a *global* feature, in which case the process is referred to as multifractal (instead of monofractal) whose scaling can only be properly characterized using a set of exponents (Kantelhardt, 2009). Alterations in the multifractal properties of neural activity were reported in many physiological and pathological conditions such as healthy aging (Mukli et al., 2018), epilepsy (Dutta et al., 2014), Alzheimer's disease (Ni et al., 2016), and also schizophrenia (Racz et al., 2020; Slezin et al., 2007). In the current work, we implicitly treated neurophysiological signals as monofractals and thus only analyzed their global scale‐free properties, as our aim was to compare the contribution of the fractal and oscillatory components to BLP estimates. However, it appears as a promising research direction to investigate the plausible time‐varying fractal nature of brain activity in SZ, estimated purely from its scale‐free component thus avoiding the confounding effects of its oscillatory components.

## 5 CONCLUSIONS

In this study, we report on decreased delta BLP over central regions in SZ when compared to HC subjects. Separate analysis of the fractal and oscillatory components of PSD estimates indicated however that most of these differences could be attributed to alterations in broadband, scale‐free rather than oscillatory brain activity. This was also emphasized by a tendency of lower scaling exponents of both low‐ and high‐range neural activity in SZ. We found a characteristic topology of spectral exponents over the cortex, further highlighting the functional significance of scale‐free neural activity and its plausible role in schizophrenia. Our findings imply that neural mechanisms different from those producing oscillatory brain activity may also contribute to the pathophysiology of schizophrenia. Our results are hoped to facilitate further research focusing on the scale‐free/fractal aspect of brain activity in SZ along with other neuropsychiatric disorders.

## CONFLICT OF INTEREST

The authors declare that the research was carried out in the absence of any commercial or financial relationships that could be construed as a potential conflict of interest.

## AUTHOR CONTRIBUTIONS

FSR designed the study and the analysis framework, performed data analysis and visualization, wrote the first draft of the manuscript and interpreted the results. KF contributed to data visualization and interpretation of the results. OS, ZK, and AC contributed to data preparation and preprocessing. PM contributed to the statistical analyses. GCS contributed to the interpretation and discussion of the results. AE provided conceptual guidance throughout the study and contributed to the discussion of the results. All authors contributed to manuscript development, provided revisions, and gave full approval on the final version.

### PEER REVIEW

The peer review history for this article is available at https://publons.com/publon/10.1002/brb3.2047.

## Supporting information

Table S1‐S3

## Data Availability

In this study, datasets of an openly available database were analyzed. The data that support the findings of this study are openly available in RepOD at http://dx.doi.org/10.18150/repod.0107441.

## Appendix 1 Separating the scale‐free component of composite signals

Scale‐free (or *fractal*) time series express self‐affinity, meaning that their statistical distribution remains unchanged when resampled at different time‐scales (Mandelbrot & Van Ness, 1968). This relationship for a scale‐free time series *f* (*t*) can be expressed as

(1) $$f_{h}\left(t\right)≜h^{H}f\left(t\right)$$

where $f_{h}\left(t\right)$is the resampled fractal time series, *h* > 0 is the resampling factor, and *H* is called the Hurst exponent (Eke et al., 2000; Mandelbrot & Van Ness, 1968). This equation implies that if the fractal time series $f\left(t\right)$ is resampled by factor *h* yielding $f_{h}\left(t\right)$, then $f_{h}\left(t\right)$ has the same statistical distribution as $f\left(t\right)$ scaled by the factor $h^{H}$. When applying the Fourier transformation, this self‐affine property will manifest as the frequency scaling property expressed as

(2) $$F_{h}\left(\omega \right)=h^{H}F\left(\omega \right)$$

where $F\left(\omega \right)$ and $F_{h}\left(\omega \right)$ are the amplitudes at angular frequencies ω for $f\left(t\right)$ and $f_{h}\left(t\right)$, respectively. Similarly, (2) implies that the amplitude of the resampled power spectrum is equal to that of the original power spectrum rescaled by $h^{H}$. It is important to highlight that this property only holds for scale‐free processes where the spectral power follows a power‐law distribution, that is, the squared amplitude is inversely proportional to the frequency according to a power‐law function with scaling exponent β (Eke et al., 2000). This can be expressed as

(3) $${\left|F\left(\omega \right)\right|}^{2}\propto c\times \omega^{‐\beta }$$

where c is a constant (Eke et al., 2000). The power spectrum of such time series follows a straight line with slope −*β* when visualized on double logarithmic axes.

Electrophysiological neural signals such as EEG are composed of both fractal and oscillatory components (He et al., 2010; Wen & Liu, 2016) that can be modeled by a simple additive model (without considering noise) as

(4) $$y\left(t\right)=f\left(t\right)+x\left(t\right)$$

where $y\left(t\right)$ is the neurophysiological signal and $f\left(t\right)$ and $x\left(t\right)$ mark the fractal and oscillatory components, respectively (Wen & Liu, 2016). Since $x\left(t\right)$ by definition is periodic and narrow‐banded, its power spectrum is non‐zero only at its characteristic frequencies; however, in rescaled versions of $x\left(t\right),$ the power is redistributed away from the original characteristic frequencies by an offset that depends on the rescaling factor (Wen & Liu, 2016). On the other hand, based on (2) and (3) the distribution of the spectral power of fractal time series (or the fractal component of a composite time series) is unaffected by resampling and yields the same distribution rescaled by $h^{H}$. Furthermore, by resampling the original fractal time series with pairwise factors comprising of a non‐negative scaling factor *h* and its reciprocal 1/*h*, the geometric mean of their auto‐power spectra returns the original power distribution (Wen & Liu, 2016; Yamamoto & Hughson, 1991, 1993). Conversely, in the case of a periodic signal this procedure will yield a power spectrum that is zero for all frequencies. The exceptions are those cases where the characteristic frequency is a common multiple of the rescaling factor *h* and its reciprocal 1/h; however, this case can be avoided with high probability by the use of multiple non‐integer rescaling factor pairs and then taking the median of power over all *h* for each frequency. Based on these principles, the fractal power spectrum of a mixed signal can be separated from the original (mixed) power spectrum, while a reasonable estimation of the power spectrum of the oscillatory component can be acquired by subtracting the fractal power spectrum from the mixed power spectrum (Wen & Liu, 2016; Yamamoto & Hughson, 1991). The above‐described procedure is termed Irregular‐Resampling Auto‐Spectral Analysis (IRASA, Wen and Liu (2016)) that is an improved version of the Coarse Graining Spectral Analysis (CGSA) method (Yamamoto & Hughson, 1991) of the same purpose, that is, separating scale‐free and oscillatory components of composite signals. Further mathematical details of IRASA and its advantages over CGSA are found in the original article (Wen & Liu, 2016).
