# Supplementary material for: Separating scale‐free and oscillatory components of neural activity in schizophrenia
Source: Brain Behav. 2021 Feb 3;11(5):e02047. doi: 10.1002/brb3.2047 (PMC8119820; doi:10.1002/brb3.2047)
Supplement: Supplementary file 1 — Table S1‐S3 [file BRB3-11-e02047-s001.docx]

**Supplementary Table S1.** Exact locations of the analyzed data segments.

| **Healthy** | | |  | **Schizophrenia** | | |
| --- | --- | --- | --- | --- | --- | --- |
| subject ID | start (s) | end (s) |  | subject ID | start (s) | end (s) |
| ’h01’ | 549 | 614.536 |  | ’s01’ | 560 | 625.536 |
| ’h02’ | 495 | 560.536 |  | ’s02’ | 760 | 825.536 |
| ’h03’ | 404 | 469.536 |  | ’s03’ | 644 | 709.536 |
| ’h04’ | 526 | 591.536 |  | ’s04’ | 910 | 975.536 |
| ’h05’ | 521 | 586.536 |  | ’s05’ | 535 | 600.536 |
| ’h06’ | 677 | 742.536 |  | ’s06’ | 489 | 554.536 |
| ’h07’ | 602 | 667.536 |  | ’s07’ | 124 | 189.536 |
| ’h08’ | 351 | 416.536 |  | ’s08’ | 227 | 292.536 |
| ’h09’ | 493 | 558.536 |  | ’s09’ | 573 | 638.536 |
| ’h10’ | 757 | 822.536 |  | ’s10’ | 734 | 799.536 |
| ’h11’ | 606 | 671.536 |  | ’s11’ | 1081 | 1146.536 |
| ’h12’ | 300 | 365.536 |  | ’s12’ | 509 | 574.536 |
| ’h13’ | 408 | 473.536 |  | ’s13’ | 108 | 173.536 |
| ’h14’ | 437 | 502.536 |  | ’s14’ | 823 | 888.536 |

# 1 Effect of window size on beta

## 1.1 Stationarity of EEG time series

In this study, we used segments of length 2^14^ data poins (approximately 65 seconds), which is considerably longer than those of 3 or 10 seconds used in previous studies utilizing the IRASA method (Kolvoort, Wainio-Theberge, Wolff, & Northoff, 2020; Muthukumaraswamy & Liley, 2018; Wen & Liu, 2016). This raises the question if electrophysiological recordings could be considered stationary over this duration. Therefore, we performed augmented Dickey-Fuller (ADF) tests on all time series to assess their stationarity. All time series analyzed in this study were rejected by the ADF tests, confirming that the investigated time series were unlikely to be realizations of a nonstationary process with a unit root.

## 1.2 Effect of window size on beta

To explicitly test the effect of window length on the estimation of $\beta$, we re-evaluated the EEG time sieres utilizing a sliding window technique with window sizes 2.5, 5 und 10 seconds and a stepsize of 0.5 second. Specifically, we divided each time series into overlapping segments of 2.5, 5 and 10 seconds with 0.5 second displacement and estimated $\beta_{lo}$ and $\beta_{hi}$ from each segment separately. For each window size we analyzed 100 consecutive segments. Spectral slopes were estimated with same input parameters as described in the main text. For each time series, its spectral slopes obtained when utilizing the entire signal ($\beta_{orig}$) were compared with the corresponding population of slopes obtained at various window lengths ($\beta_{window}$). In that, the null hypothesis that the $\beta_{orig}$ values came from the same distribution of the corresponding $\beta_{window}$ values was rejected at the level $\alpha=0.05$ if the given $\beta_{orig}$ was found outside the [2.5%; 97.5%] quantile range obtained from the $\beta_{window}$ population. The proportions of cases (both groups combined) where the spectral slopes obtained using the entire signal were found similar to those acquired using smaller window sizes are summarized in **Supplementary Table S2**. The high fraction of similar spectral slopes indicates that utilizing the entire signal for the estimation of $\beta$ did not yield values substantially different from those obtained at smaller window sizes.

**Supplementary Table S2.** Proportions of cases in which different window sizes yielded spectral slopes similar to those obtained when using the entire signal.

|  | **2.5-second window** | | **5-second window** | | **10-second window** | |
| --- | --- | --- | --- | --- | --- | --- |
| channel | $\beta_{lo}$ | $\beta_{hi}$ | $\beta_{lo}$ | $\beta_{hi}$ | $\beta_{lo}$ | $\beta_{hi}$ |
| Fp2 | 100% | 100% | 100% | 100% | 100% | 100% |
| F8 | 100% | 100% | 100% | 100% | 100% | 100% |
| T4 | 100% | 100% | 100% | 100% | 100% | 100% |
| T6 | 100% | 100% | 100% | 100% | 96.43% | 100% |
| O2 | 100% | 100% | 100% | 96.43% | 100% | 96.43% |
| Fp1 | 100% | 100% | 100% | 100% | 100% | 100% |
| F7 | 100% | 100% | 100% | 100% | 100% | 100% |
| T3 | 100% | 100% | 100% | 100% | 100% | 96.43% |
| T5 | 100% | 100% | 100% | 96.43% | 100% | 96.43% |
| O1 | 100% | 100% | 100% | 100% | 100% | 100% |
| F4 | 96.43% | 100% | 96.43% | 100% | 96.43% | 100% |
| C4 | 96.43% | 100% | 96.43% | 100% | 100% | 100% |
| P4 | 100% | 100% | 100% | 96.43% | 100% | 96.43% |
| F3 | 100% | 100% | 100% | 100% | 100% | 100% |
| C3 | 100% | 100% | 100% | 100% | 100% | 100% |
| P3 | 100% | 100% | 100% | 100% | 100% | 100% |
| Fz | 100% | 100% | 100% | 100% | 100% | 100% |
| Cz | 100% | 100% | 100% | 100% | 100% | 100% |
| Pz | 100% | 100% | 100% | 100% | 100% | 100% |

# 2. True bimodality of the power spectrum

## 2.1 Effects of the range of resampling factors

The bimodal (or multimodal) nature of neurophysiological signals is a commonly observed phenomenon (Eke, Herman, & Hajnal, 2006; He, Zempel, Snyder, & Raichle, 2010; Mukli, Nagy, Racz, Herman, & Eke, 2018; Nagy, Mukli, Herman, & Eke, 2017), however this property is immensely difficult to assess in practice. Although the IRASA procedure provides a tool for reducing the bias from oscillatory components when estimating $\beta$, some additional remarks must be made when applying it to assess multiple betas of different frequency regimes. Essentially, IRASA removes oscillatory spikes from the power spectrum by relocating them proportionally to the resampling factor, then taking the median over the power spectra of the resampled time series. This works efficiently for well-defined spikes such as those produced by line noise at 50 or 60 Hz even with a limited range of resampling factors. However, in case of larger oscillatory components – such as the alpha peak – that may span multiple frequencies a broader range of resampling factors is preferred, otherwise the peak will be rather ’smeared’ across the fractal power spectrum due to insufficient relocation, potentially biasing the estimation of $\beta$. Although this may be optimal for signals with unimodal spectra, in case of a bimodal spectrum a broader range of rescaling factors comes at the expense of the breakpoint (separating the two regimes with different spectral slopes) being ’smoothened out’ (Wen & Liu, 2016). In other words, there is a tradeoff between a complete elimination of oscillatory spikes and a well-defined breakpoint. Additionally, it has to be noted that in case of (low/high- or band-pass) filtered signals, the resampling procedure also relocates filter boundaries, limiting the frequency range in which $\beta$ can be estimated (Wen & Liu, 2016). For example, upsampling the signal by a factor of 1.5 would relocate a cutoff frequency of 45 Hz to 30 Hz in the power spectrum of the resampled signal. This could not only bias the estimation of $\beta$ but in some cases even yield the false appearance of a bimodal spectrum.

## 2.2 Assessing true bimodality of the power spectra

Although the difference in $\beta_{lo}$ and $\beta_{hi}$ was found significantly different from 0 in most cases, this result in itself does not confirm if a bimodal model indeed provides a better representation of the data than a unimodal model. In order to explicitly test this hypothesis, we compared Goodness of Fit (GoF) statistics of uni- (i.e. a single linear function was fitted on the entire power spectrum) and bimodal fits similarly to as described in the supplementary material of Mukli et al. (2018). In that, GoF was characterized by the sum of squared errors (SSE) in case of uni- (${SSE}_{uni}$) and bimodal (${SSE}_{bi}$) evaluation schemes. The test statistic T was defined according to

| $T=\frac{\frac{{SSE}_{uni}-{SSE}_{bi}}{p_{bi}-p_{uni}}}{\frac{{SSE}_{uni}}{n-(p_{bi}+1)}},$ | (S1) |
| --- | --- |

in which $p_{bi}$ and $p_{uni}$are the number of free parameters in the model, and $n$ is the number of examples used for fitting the model. The free parameters of the unimodal model were the frequency limits (1 and 30 Hz, thus $p_{uni}=2$), while the additional border frequency of 13 Hz yielded $p_{bi}=3$. The linear was fitted on $n=1901$ frequency components. Since$T$ follows an F distribution, an F test can be utilized in order to verify if the bimodal model provides a better fit for the data. Detailed results for all channels are shown in **Supplementary Table S3**. These results indicate that a bimodal model indeed provided a better fit than the unimodal for almost all cases, while those cortical locations where the succes rate was lower than 100% correspond well to those with non-significant differences between $\beta_{lo}$ and $\beta_{hi}$ (shown in **Figure 3**).

**Supplementary Table S3.** Fraction of time series passing the F test for bimodality.

| Channel | Healthy | Schizophrenia |
| --- | --- | --- |
| Fp2 | 100.0% | 92.86% |
| F8 | 85.71% | 92.86% |
| T8 | 100.0% | 92.86% |
| P8 | 100.0% | 100.0% |
| O2 | 100.0% | 100.0% |
| Fp1 | 92.86% | 100.0% |
| F7 | 92.86% | 100.0% |
| T7 | 100.0% | 100.0% |
| P7 | 100.0% | 100.0% |
| O1 | 100.0% | 100.0% |
| F4 | 92.86% | 100.0% |
| C4 | 100.0% | 100.0% |
| P4 | 100.0% | 100.0% |
| F3 | 100.0% | 100.0% |
| C3 | 100.0% | 100.0% |
| P3 | 100.0% | 100.0% |
| Fz | 100.0% | 100.0% |
| Cz | 100.0% | 100.0% |
| Pz | 100.0% | 100.0% |

# 3. Validation of the results

With the previously discussed considerations in mind, in the current study we set the resampling factor *h* in the range of 1.05 and 1.5. With this setup the effective frequency range from where $\beta$ could be reliably estimated was as broad as 1-30 Hz as defined by filtering characteristics (0.5-45 Hz). We found that this frequency range is required for reliable estimation of $\beta_{lo}$ and $\beta_{hi}$ with a prominent breakpoint (i.e. the power spectrum consisted of two well-defined regimes instead of appearing ’bended’). However, it also has to be noted that with these settings some residual alpha activity may remain in the power spectrum, which may potentially bias the estimation of $\beta_{lo}$ and $\beta_{hi}$ to some extent, as well as fractal BLP. Therefore, we re-evaluated the dataset with using 25 evenly distributed resampling factors spanning from 1.05 to 2. As these settings reduce the effective frequency range to [1 Hz; 20 Hz], in this analysis we only focused on $\beta_{lo}$ and BLP in the delta, theta and alpha bands. This analysis pipeline yielded results similar to those described in the main text. Specifically, mixed delta BLP was found significantly lower in the SZ group over C3 (*p*=0.0371, corrected, AP=0.3620, ES=1.0994), however the same difference was found only marginally significant in fractal delta BLP following FDR adjustment (*p*=0.0663, corrected, AP=0.3158, ES=1.0469). When results were compared on the RSN level, we found significantly lower delta BLP in SZ over the somatomor network in both mixed (*p*=0.0035, corrected, AP=0.6384, ES=1.1832) and fractal (*p*=0.0079, corrected, AP=0.5759, ES=1.1172), but not in oscillatory spectra. In both cases, when mixed BLP was compared between groups with fractal BLP as a covariate, the previously observed differences in mixed BLP were rendered non-significant (*p*>0.05 in both cases), indicating that they were at least in part consequences of reduction of fractal BLP in SZ. No significant group-level differences were found in spectral slope nor oscillatory BLP in any frequency bands.

# References

Eke, A., Herman, P., & Hajnal, M. (2006). Fractal and noisy CBV dynamics in humans: influence of age and gender. *J Cereb Blood Flow Metab, 26*(7), 891-898. doi:10.1038/sj.jcbfm.9600243

He, B. J., Zempel, J. M., Snyder, A. Z., & Raichle, M. E. (2010). The temporal structures and functional significance of scale-free brain activity. *Neuron, 66*(3), 353-369. doi:10.1016/j.neuron.2010.04.020

Kolvoort, I. R., Wainio-Theberge, S., Wolff, A., & Northoff, G. (2020). Temporal integration as "common currency" of brain and self-scale-free activity in resting-state EEG correlates with temporal delay effects on self-relatedness. *Hum Brain Mapp*. doi:10.1002/hbm.25129

Mukli, P., Nagy, Z., Racz, F. S., Herman, P., & Eke, A. (2018). Impact of Healthy Aging on Multifractal Hemodynamic Fluctuations in the Human Prefrontal Cortex. *Frontiers in Physiology, 9*. doi:10.3389/fphys.2018.01072

Muthukumaraswamy, S. D., & Liley, D. T. J. (2018). 1/f electrophysiological spectra in resting and drug-induced states can be explained by the dynamics of multiple oscillatory relaxation processes. *Neuroimage, 179*, 582-595. doi:10.1016/j.neuroimage.2018.06.068

Nagy, Z., Mukli, P., Herman, P., & Eke, A. (2017). Decomposing multifractal crossovers. *Frontiers in Physiology, 8*(JUL), 533. doi:10.3389/fphys.2017.00533

Wen, H. G., & Liu, Z. M. (2016). Separating Fractal and Oscillatory Components in the Power Spectrum of Neurophysiological Signal. *Brain Topography, 29*(1), 13-26. doi:10.1007/s10548-015-0448-0
